# Evolution, diversification, and expression of KNOX proteins in plants

**DOI:** 10.3389/fpls.2015.00882

**Published:** 2015-10-23

**Authors:** Jie Gao, Xue Yang, Wei Zhao, Tiange Lang, Tore Samuelsson

**Affiliations:** ^1^Key Laboratory of Tropical Forest Ecology, Xishuangbanna Tropical Botanical Garden, Chinese Academy of SciencesMenglun, China; ^2^Department of Life Sciences, Jilin Agricultural UniversityJilin, China; ^3^State Key Laboratory of Systematic and Evolutionary Botany, Institute of Botany, Chinese Academy of SciencesBeijing, China; ^4^Department of Medical Biochemistry and Cell Biology, Institute of Biomedicine, Sahlgrenska Academy at University of GothenburgGothenburg, Sweden

**Keywords:** bioinformatics, green algae, *Glycine max*, KNOX, plant evolution

## Abstract

The KNOX (KNOTTED1-like homeobox) transcription factors play a pivotal role in leaf and meristem development. The majority of these proteins are characterized by the KNOX1, KNOX2, ELK, and homeobox domains whereas the proteins of the KNATM family contain only the KNOX domains. We carried out an extensive inventory of these proteins and here report on a total of 394 KNOX proteins from 48 species. The land plant proteins fall into two classes (I and II) as previously shown where the class I family seems to be most closely related to the green algae homologs. The KNATM proteins are restricted to Eudicots and some species have multiple paralogs of this protein. Certain plants are characterized by a significant increase in the number of *KNOX* paralogs; one example is *Glycine max*. Through the analysis of public gene expression data we show that the class II proteins of this plant have a relatively broad expression specificity as compared to class I proteins, consistent with previous studies of other plants. In *G. max*, class I protein are mainly distributed in axis tissues and KNATM paralogs are overall poorly expressed; highest expression is in the early plumular axis. Overall, analysis of gene expression in *G. max* demonstrates clearly that the expansion in gene number is associated with functional diversification.

## Introduction

Leaves are responsible for photosynthesis and transpiration and are highly diverse within the plant kingdom. In angiosperms, in particular, leaves vary remarkably in shape and size, making them an attractive system for the study of the evolution of form. Leaf development involves many gene families (Efroni et al., 2010) and is a very complex process, including the initiation of a leaf primordia and establishment of leaf polarity. Leaf development may depend heavily on the activities of homeodomain (HD) KNOTTED-like homeobox transcription factors (*KNOX*), which control the formation and maintenance of the shoot apical meristem (SAM) (Smith et al., 1992; Hake et al., 2004; Hay and Tsiantis, 2010; Tsuda and Hake, 2015). The *KNOX* genes belong to a large family of transcription factors called homeobox genes, which possess a conserved DNA-binding domain (homedomain) that controls growth and pattern formation during development in many organisms, including plants, insects, and mammals (Mukherjee et al., 2009; Furumizu et al., 2015). *KNOX* genes are generally distinguished by four characteristic domains: KNOX1, KNOX2, ELK, and KN HDs (Vollbrecht et al., 1991; Bürglin, 1997, 1998). However, the *KNATM* genes (Magnani and Hake, 2008) contain the KNOX1 and KNOX2 domains but lack the ELK and HDs. Genetic analyses identify a function for KNATM in both transcriptional regulation and leaf proximal-distal patterning (Magnani and Hake, 2008; Peng et al., 2011).

The first *KNOX* gene to be identified in plants was KNOTTED1 (kn1) in maize (Vollbrecht et al., 1991). Following this discovery, a number of studies on the KNOX proteins have been carried out in model and non-model plants. The functions of KNOX have been studied extensively in *Arabidopsis*, rice, and tomato (Long et al., 1996; Postma-Haarsma et al., 1999; Nagasaki et al., 2001; Douglas et al., 2002; Belles-Boix et al., 2006; Jasinski et al., 2007; Ragni et al., 2008). Based on sequence similarity, intron position, expression pattern, and phylogenetic analysis, the *KNOX* genes can be divided into two subclasses: KNOX I and KNOX II (Kerstetter et al., 1994; Bharathan et al., 1997; Mukherjee et al., 2009; Furumizu et al., 2015). The functions of class I *KNOX* genes have been intensively studied. In *Arabidopsis* the KNOX I class contains four genes: *SHOOTMERISTMELESS* (*STM*), *KNAT 1, KNAT 2*, and *KNAT 6*. The gene *STM* is essential for the formation and maintenance of the shoot apical meristem (SAM). *KNAT 1* and *KNAT 6* contribute to SAM function and inflorescence development (Byrne et al., 2002; Douglas et al., 2002; Venglat et al., 2002; Ragni et al., 2008), while *KNAT 2* regulates flower patterning (Dockx et al., 1995; Pautot et al., 2001; Li et al., 2012a). The *KNOX I* proteins form heterodimers with other HDs (e.g., BEL-like homedomain) in the TALE superclass and regulate downstream gene activities with different combinations of KNOX/BLH transcription factors (Arnaud and Pautot, 2014). In contrast to the well- studied class I *KNOX* genes, the functions of class II *KNOX* genes remain largely unresolved. Among class II *KNOX* genes, *KNAT7* has received the most attention and is known to play a role in the transcriptional network regulating secondary cell wall biosynthesis (Li et al., 2011, 2012b; Gong et al., 2014; Liu et al., 2014). Additionally, *KNAT 3* may regulate abscisic acid (ABA) responses during germination and early seeding development in *Arabidopsis* (Kim et al., 2013). *KNOX I* and *KNOX II* genes perform non-redundant functions in concert to control the development of all above-ground organs of the *Arabidopsis* sporophyte (Furumizu et al., 2015).

However, little is known about the features of *KNOX* genes across Viridiplantae, despite extensive studies within selected plant species (Bharathan et al., 1999; Champagne and Ashton, 2001; Guillet-Claude et al., 2004; Di Giacomo et al., 2008; Testone et al., 2012). Thus, a complete survey of *KNOX* genes across Viridiplantae is necessary. Herein we present a systematic inventory of plant KNOX proteins in a total of 48 plant genomes, ranging from Chlorophyta to higher plants, adding to the present understanding of the evolution of this family. We found that the KNOX proteins in green algae formed a distinct clade in our phylogenetic analyses but are most similar to the class I KNOX proteins of land plants. Proteins belonging to the KNATM subfamily within KNOX arose at a later stage during the plant evolution and are restricted to eudicots. The class II KNOX proteins are much more conserved in sequence as compared to class I and seem to have been under stronger purifying selection. The *KNOX* genes have experienced two major events of expansion during the evolution of plants, and some species underwent dramatic increases in KNOX paralogs. One such example is the soybean *G. max* and using an extensive expression dataset of this plant we were able to examine in detail tissue specificity and its relationship to the expansion of KNOX genes.

## Materials and methods

### *KNOX* gene identification and bioinformatics analysis

The amino acid sequences of *Arabidopsis thaliana KNOX* genes were retrieved from The *Arabidopsis* Information Resource (TAIR, http://www.arabidopsis.org) and used as queries to search against other plant genome databases with BlastP and tBlastN programs (default parameters). We obtained plant protein sequences from Phytozome and Genbank, including sequences of green and red algae. We analyzed all protein sequences with HMMER3.0 (http://hmmer.janelia.org/, default parameters) and with rpsblast (Altschul et al., 1997, default parameters) in order to identify domains characteristic of KNOX proteins.

### Phylogenetic analysis

We aligned amino acid sequences using either the Clustal X 1.83 software (Thompson et al., 1997) or Clustal Omega, using default parameters (Sievers et al., 2011). Alignments were manually edited using either BioEdit (Hall, 1999) or Jalview (Waterhouse et al., 2009). We constructed phylogenetic trees based on the amino acid sequences of the conserved domains of *KNOX* using a maximum likelihood (ML) method in PHYML (Guindon and Gascuel, 2003); neighbor-joining (NJ) in MEGA5 (Tamura et al., 2011) or ClustalW2 (Thompson et al., 1997) with 1000 bootstrap replicates; or Bayesian methods in MrBayes with prset aamodelpr = mixed and 2,000,000 generations. The optimal amino acid substitution model was calculated by ModelGenerator v0.84 (Keane et al., 2006) with the optimal model of “JTT+G.”

### Evolutionary rate and selection evaluation

To examine evolutionary rates, we calculated the values of both *dN* (non-synonymous substitution rate) and *dS* (synonymous substitution rate) of the two gene classes using the Kumar model (Kimura 2-para) with MEGA5 (Goldman and Yang, 1994; Yang, 2007). We also used the CODEML program in the PAML software package to test the branch models (Yang, 2007). The branch models of CODEML were used to estimate ω (= *dN*/*dS*) under two assumptions: a one-ratio model that assumed the same ω-value for all branches, and a two-ratio model that allowed the ω-value vary between the different branches. Likelihood ratio tests (LRTs) were performed by comparing twice the difference in log-likelihood values between pairs of the models using a *X*^2^ distribution (Yang and Nielsen, 2000).

### Expression analysis

To investigate the expression patterns of the *Physcomitrella patens KNOX* gene family under normal condition, we cultured *P. patens* plants in BCDATG solidified medium (Nishiyama et al., 2000) at 25°C under an 18/6-h light/dark cycle for ~6 months. We isolated total RNA from the rhizoids, stems, and leaves of the *P. patens* plants. To investigate the expression patterns of *KNOX* genes in *Selaginella*, we cultivated *Selaginella* plants in potting soil for 2 years. We isolated total RNA from the roots, stems, and leaf tissues of the *Selaginella* plants. For both *Physcomitrella* and *Selaginella*, we isolated total RNA with an Aurum Total RNA Kit (Bio-Rad, Hercules, CA, USA). We treated the total RNA with RNase-free DNase I (Promega) and reverse transcribed into cDNA using an RNA PCR Kit (AMV) version 3.0 (TaKaRa). Based on the multiple sequence alignment of *KNOX* gene sequences, we designed specific PCR primers (Supplementary Table 1). We optimized PCR conditions to include an initial denaturation of 3 min at 94°C, followed by 30 cycles of 30 s at 94°C, 40 s at 65°C, and 1 min at 72°C with a final extension of 3 min at 72°C. In all PCR analyses, we used actin genes (*Selaginella pallescens* Genbank: FN600550.1; and *Act5* gene from *P. patens*, Genbank: AY382284.1) as internal controls for amplification. We analyzed PCR products from each sample using a 1% agarose gel and validated them with DNA sequencing.

To investigate the expression patterns in other species, we obtained publically available expression data. Data for *Arabidopsis* and *Populus* were from AtGenExpress (Schmid et al., 2005) and poplar eFP (Wilkins et al., 2009), respectively. We obtained expression data for *Zea mays* from the Plant Expression Database (PLEXdb, http://www.plexdb.org/) (Sekhon et al., 2009; Dash et al., 2012). For rice, we used RNA-seq data from the Michigan State University (MSU) Rice Genome Annotation (http://rice.plantbiology.msu.edu) databases. We also analyzed a large dataset of gene expression in the soybean *G. max* generated with RNA-Seq (the Harada-Goldberg soybean seed LCM dataset) from a wide variety of tissues, 203 samples, and 346 sequencing runs. We aligned the RNA-Seq reads with bwa (Burrows-Wheeler Aligner, Li and Durbin, 2009) against a database of mRNAs including relevant coding sequences of KNOX proteins. The bwa aligner, version 0.7.10, was used with default parameters, including a maximum mismatch frequency 4% of read length. We calculated logodds ratios for each *KNOX* gene as log (*m* ^*^ 100000/*t*), where *m* is the number of uniquely mapped reads and *t* is the total number of reads mapping to the transcriptome. Finally, we generated heatmaps from logodds ratios using the “pheatmap” package in R (http://www.r-project.org/) with scale = “none” and default parameters.

## Results

### *KNOX* proteins are present in all investigated land plant species, as well as in specific phyla of green algae

**Figure 1:**
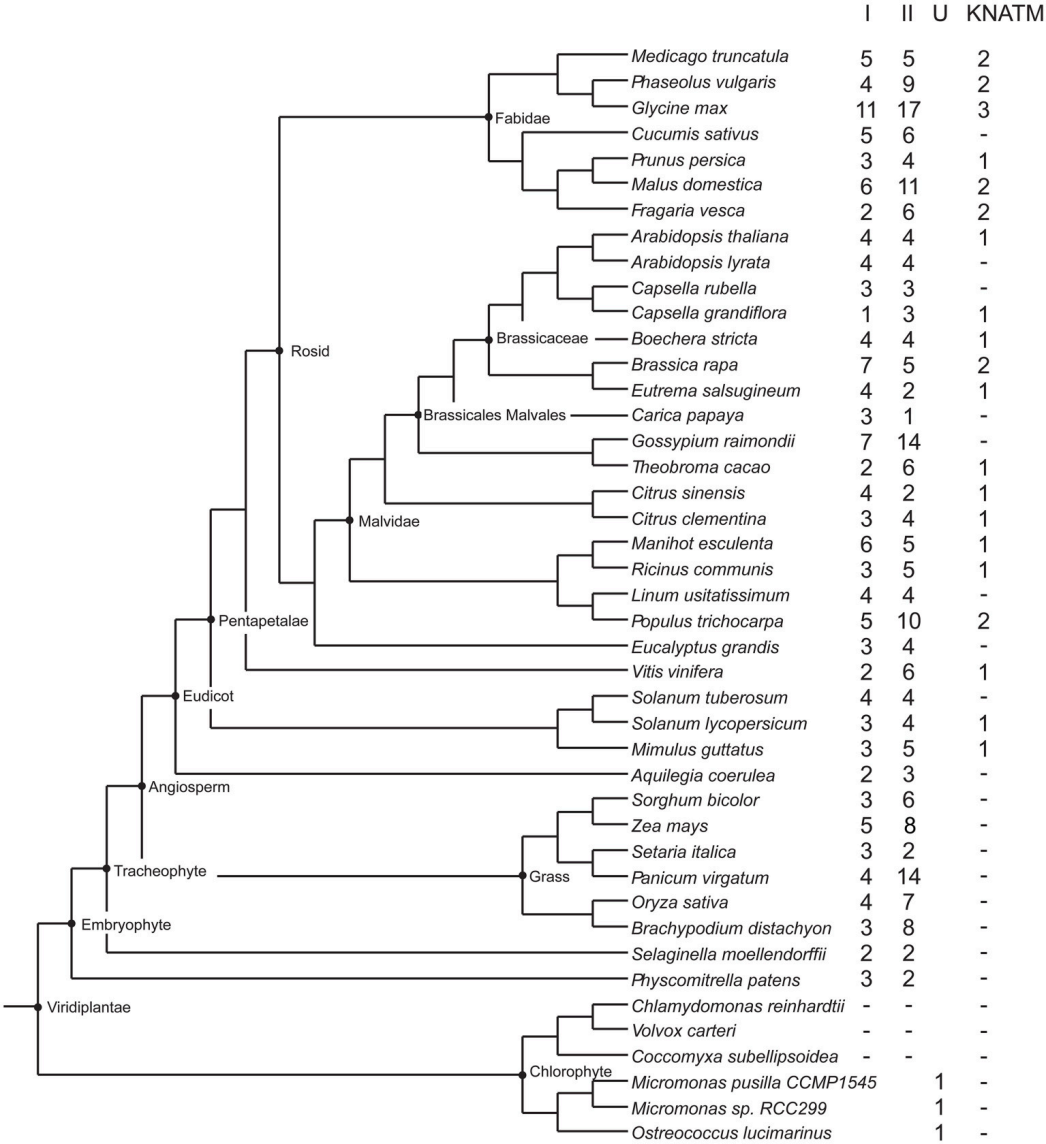
**Overview of plants and number of KNOX homologs**. The schematic tree is based on the tree available at http://phytozome.jgi.doe.gov/pz/portal.html. Columns show number of homologs identified being members of class I, class II, unclassified (green algae), and KNATM, respectively.

A number of plant protein sequences available at Phytozome (http://phytozome.org/), as well as green and red algae from the NCBI GenBank, were analyzed with respect to their protein domain structure. A total of 48 species were searched with respect to domains characteristic of KNOX proteins, i.e., the KNOX1, KNOX2, ELK, and Homeobox_KN domains, as further detailed in the Materials and Methods Section (All the species are listed in the alignment file in Supplementary Data Sheet 1). Out of a total of 394 proteins, we identified 358 proteins with all four domains, and 36 proteins with only the KNOX1 and KNOX2 domains (Figure 1). We found proteins with all four domains in many green algae (*Ostreococcus lucimarinus, Ostreococcus tauri, Bathycoccus prasinos*, and *Micromonas*, belonging to prasinophytes and *Acetabularia acetabulum* of Ulvophyceae) and in all land plants we examined. We attempted to identify KNOX genes in red algae using HMMs, based on the KNOX1 and KNOX2 domains identified in green algae, but our results were negative.

The bryophyte *P. patens* and the lycophyte *S. moellendorffii* contained only five and four KNOX proteins, respectively. In contrast, the KNOX protein family was greatly expanded in angiosperms. The number of KNOX genes varies dramatically among eudicots and ranges from a limited number of proteins in *Campsis grandiflora* (5) and *Linum usitatissimum* (8) to an extensive expansion in *G. max* (31). We found KNATM family KNOX proteins (i.e., proteins with only KNOX1 and KNOX2 domains) exclusively in eudicots and we noted that there are species that contain more than one homolog of the KNATM protein. In particular, we identified three KNATM paralogs in *G. max* and two paralogs in *Medicago truncatula, Proteus vulgaris, Malus domestica, Fragaria vesca, Brassica rapa*, and *P. trichocarpa* (Figure 1).

### Algae KNOX proteins are most closely related to class I homologs in higher plants

**Figure 2:**
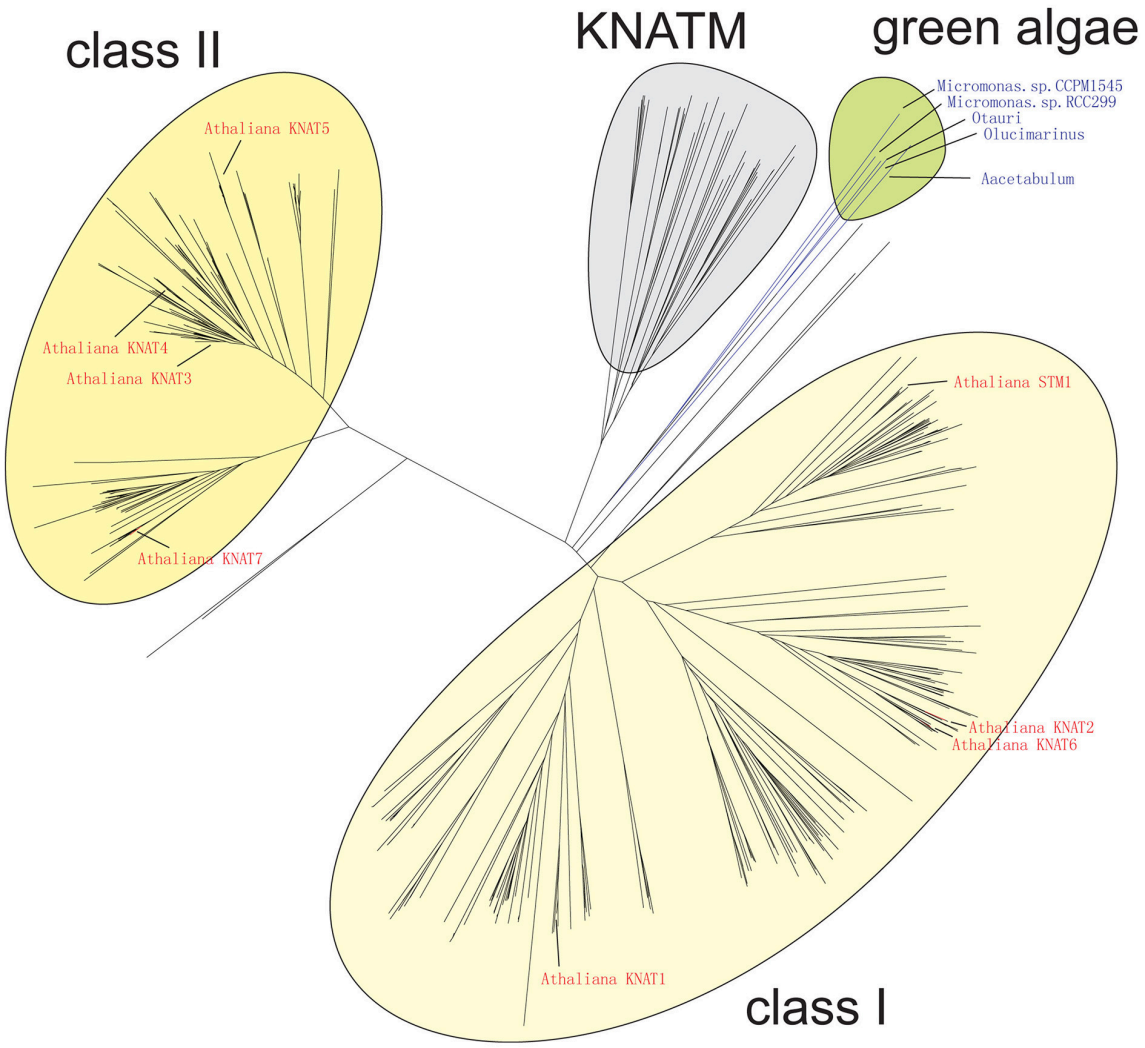
**Grouping of KNOX homologs**. A collection of 394 KNOX proteins were grouped using neighbor-joining. Sequences of *A. thaliana* and green algae are highlighted.

The result of our NJ phylogenetic analysis is shown in Figure 2 (unrooted tree) and in Supplementary Figure 1 (phylogram) (In Figure 2 most of the species names have been left out to avoid overcrowding, but these are all shown in the Supplementary Figure 1). The NJ tree shows four major clades: the KNATM, KNOX I, KNOX II, and green algae. Therefore, this analysis confirms the previously reported classification of class I and class II KNOX proteins and shows that KNATM is distinct from them (Mukherjee et al., 2009; Furumizu et al., 2015). We confirmed the relationships among *KNOX* genes observed in the NJ tree using a smaller dataset with MrBayes (Supplementary Figure 2).

**Figure 3:**
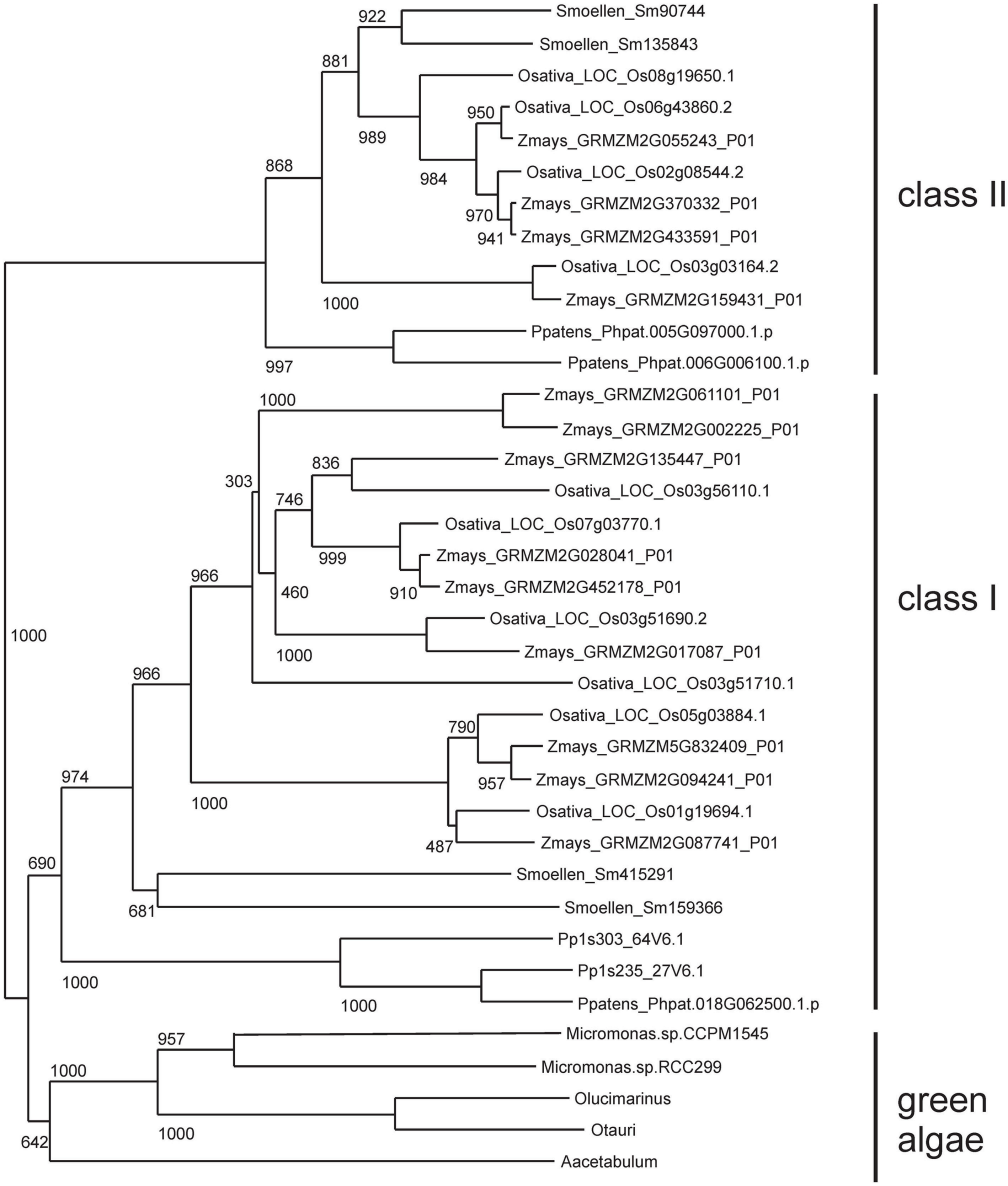
**Algae sequences group to classes I KNOX proteins**. Grouping of 37 sequences were performed with ClustalW2 using 1000 bootstrap replicates.

The Bayesian tree (Supplementary Figure 2) and NJ (Figure 3) trees suggest that the green algae sequences are most closely related to the class I KNOX proteins of higher plants. Additionally, the alignment in Supplementary Figure 3 highlights positions that are consistent with the grouping of green algae with KNOX I. Therefore, these results support the idea that class I proteins may represent the ancestral form of higher plant KNOX proteins. The KNATM proteins form a distinct group according to all phylogenetic analyses. These proteins appear to be restricted to the eudicots and probably originate from a lineage of class I or II KNOX proteins that lost their ELK and HDs. Our phylogenetic analyses did not reveal whether KNATM genes arose from class I or II genes.

### Class II KNOX proteins are much more conserved in sequence than class I proteins and were likely under purifying selection

For the analysis of the evolutionary rates, we focused on selected species, including *P. patens, S. moellindorffi, A. thaliana, P. trichocarpa, O. sativa, Z. may*, and *G. max*. KNOX I and KNOX II proteins were characterized by >29 and >52% pairwise sequence identity, respectively (Supplementary Figure 4). At the same time, the sequence identity between the two classes was very low, and an independent sample *t*-test showed that the protein sequence identities within each class were higher than that between classes (*P* < 0.0001). The sequence conservation in the different protein classes is also illustrated by the Supplementary Figures 3, 4.

**Figure 4:**
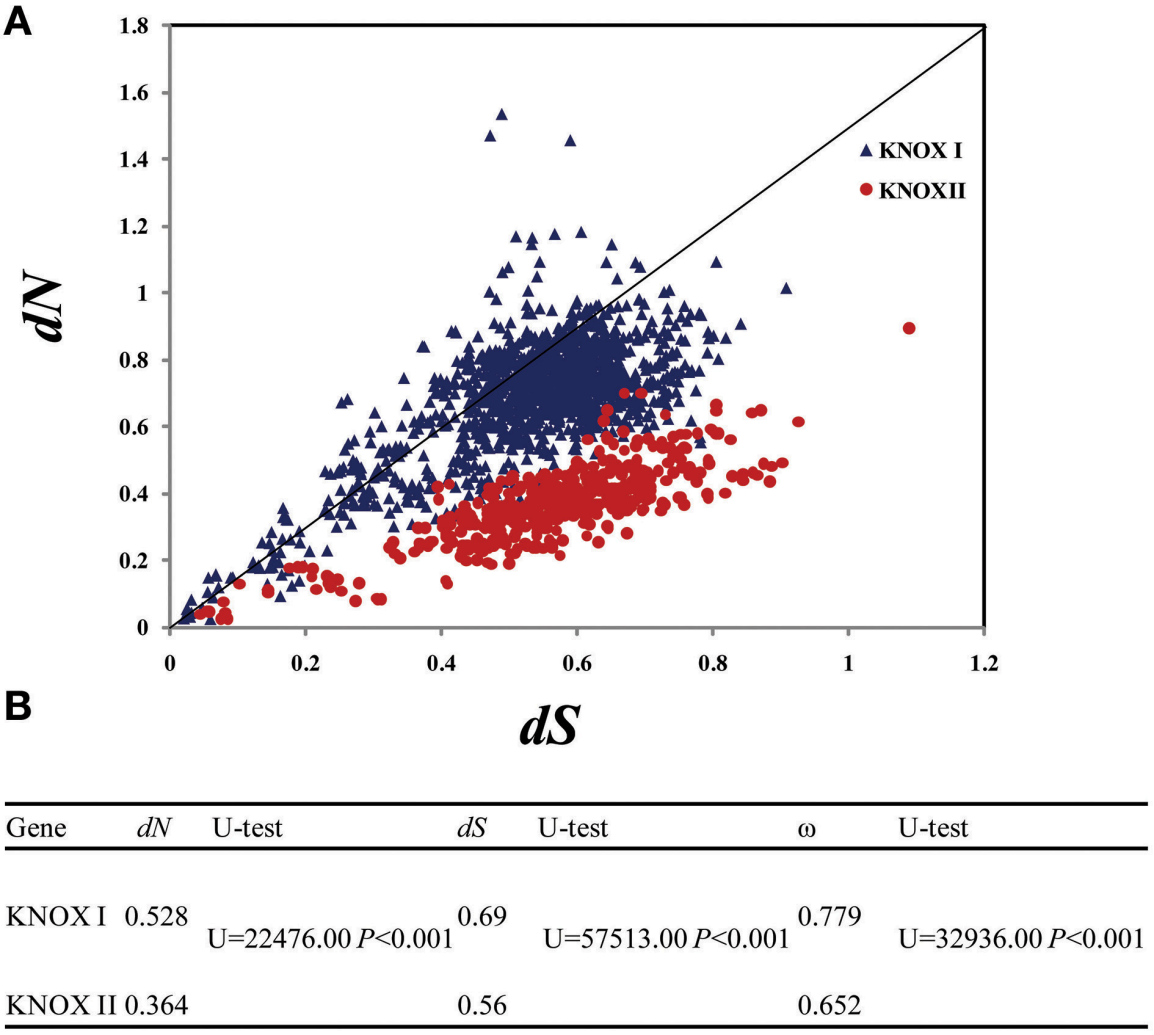
**Evolutionary rates of class I and class II ***KNOX*** genes**. The species analyzed were *Physcomitrella patens, Selaginella moellendorffii, Oryza sativa, Zea mays, Arabidopsis thaliana, Populus trichocarpa*, and *G. max*. **(A)** Evolutionary rate of the *KNOX I* and *KNOX II* genes. *dN*, non-synonymous substitution rate; *dS*, synonymous substitution rate. **(B)** Comparison of the evolutionary rage, the average values of the *KNOX I* and *KNOX II* genes, Utest: Mann-whitney Rank sum test.

To examine evolutionary rates, we calculated the values of both *dN* (non-synonymous substitution rate) and *dS* (synonymous substitution rate) of the two gene classes. *dS* varied between the *KNOX I* genes from 0.03 to 1.506 and within the *KNOX II* from 0.027 to 1.18, while *dN* ranged from 0.007 to 1.485 for the *KNOX I* genes and from 0.028 to 0.79 for *KNOX II* (Figure 4A). The average *dN*-values were small: 0.528 for *KNOX* I and 0.364 for *KNOX* II. The average *dS*-values were somewhat higher, 0.69 for *KNOX* I and 0.56 for *KNOX* II, and they were significantly different (*U* = 22, 476.00 and 57,513.00, respectively, *P* < 0.01) (Figure 4B). Both the *dN*- and *dS*-values for *KNOX* I were higher than for *KNOX* II, suggesting that these two classes of genes evolved with non-uniform evolutionary rates. The average ω (*dN*/*dS*)-values for *KNOX* I were 0.779 and 0.652 for *KNOX* II. These values of ω were significantly different (*U* = 32,936.00, *P* < 0.001; Figure 4B), suggesting that the two classes of proteins experienced different degrees of purifying selection.

We also applied another approach for testing the evolutionary rates of the *KNOX* genes by using the CODEML program in the PAML software package. In particular, we tested the branch models. We used *KNOX* genes from seven major species: *P. patens, S. moellindorffi, A. thaliana, P. trichocarpa*, and *G. max*, as well as *O. sativa* and *Z. mays*, and constructed a phylogenetic tree for PAML analysis (Supplementary Figure 5). The comparison of the one-ratio model to the two-ratio model using LRTs showed that the two-ratio model was significantly better fit than the one-ratio model (Table 1), suggesting significant differences in selective pressure between the two classes of *KNOX* genes. The mean ω-value in class II *KNOX* (0.0042) was lower than that in class I *KNOX* (0.0638), indicating that *KNOX* II genes were under stronger purifying selection than *KNOX* I (Table 1). The results of the two branch models were consistent with the analyses of ω.

**Table 1:** **Summary statistics for detection of selection using branch models of PAML**.

**Model**	**Estimates of parameters**	**lnL**	**2▵lnL**	***P***
One ratio	ω = 0.0638 for all branches	−16,796.1591		
Two ratio	ω_1_ = 0.0638 for KNOX I	−16,795.6552	1.01	<0.3
	ω_0_ = 0.0042 for KNOX II			

### Expression analysis of *KNOX* genes

To investigate the functional diversity of the *KNOX* genes, we examined the expression patterns of *Physcomitrella* and *Selaginella KNOX* genes in different tissues using PCR. The *Physcomitrella* genome contains three class I and two class II *KNOXs*. Among the three genes from class I, one was expressed in all tissues examined, one was not expressed in any of the tissues, and one was expressed in stem and leaf tissues but not in rhizoid. One of the two genes from class II was expressed in all tissues examined, but the other was not expressed in any of the tissues (Supplementary Figure 6). The *Selaginella* genome possesses two genes from each class, and all these genes were expressed in all tissues examined (Supplementary Figure 6).

**Figure 5:**
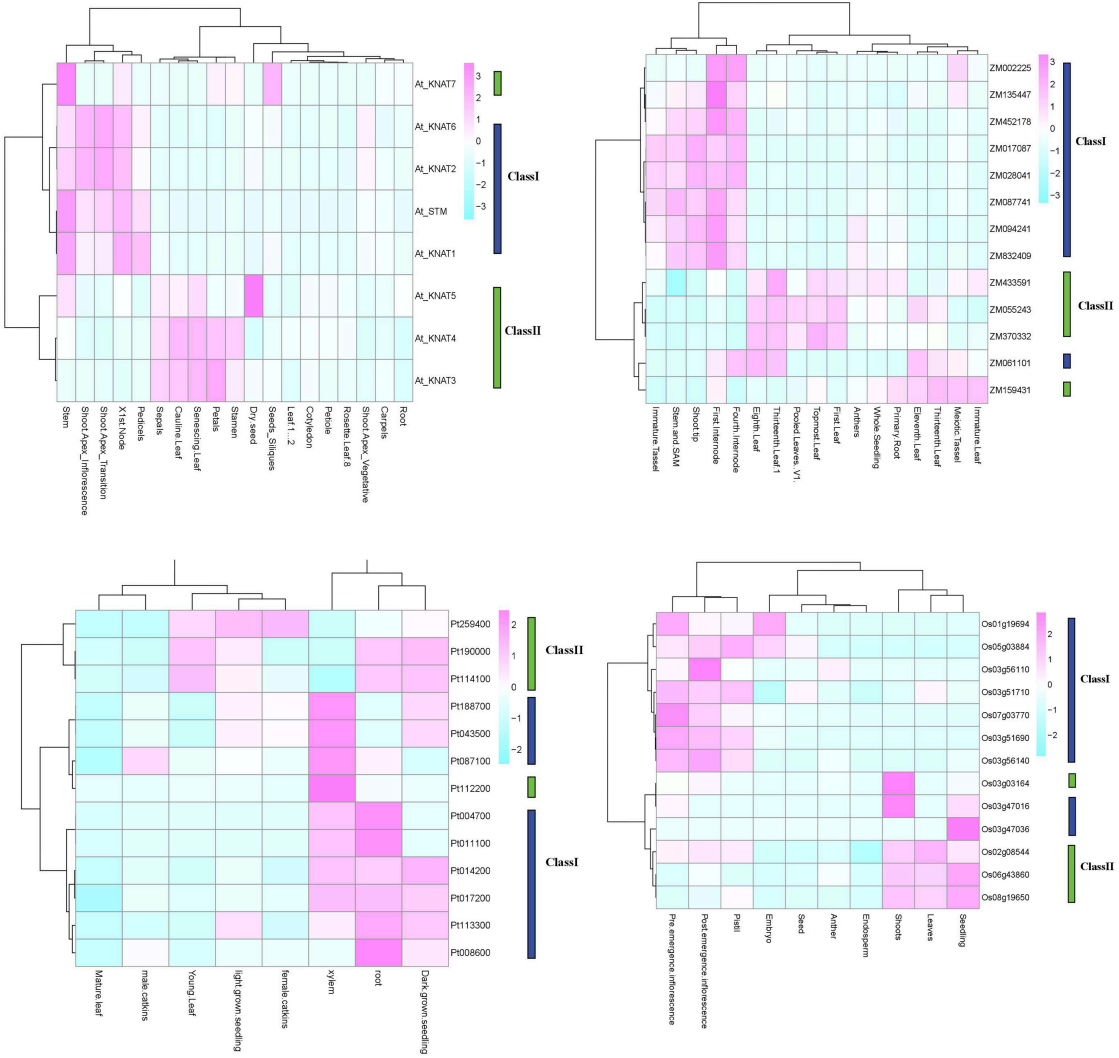
**KNOX expression in four species**. Expression profiles for *O. sativa, Z. mays, P. trichocarpa*, and *A. thaliana* are shown. Analyses are based on publically available microarray data. The *Arabidopsis* and *Populus* microarray gene expression data were obtained from AtGenExpress and poplar eFP. The public expression data in *Zea mays* were obtained from the Plant Expression Database (PLEXdb, http://www.plexdb.org/). The rice expression, we used RNA-seq data from the Michigan State University (MSU) Rice Genome Annotation (http://rice.plantbiology.msu.edu) databases.

For a set of the higher plants, we used publicly available microarray data to investigate the patterns of tissue-specific expression. The results are presented in Figure 5. In *Arabidopsis*, the class I *KNOX* genes *KNAT2/6, KNAT1*, and *STM*, had an elevated expression in inflorescence shoot apex and in node and stem apex (SAM). The *STM* and *KNAT1* genes were also expressed at higher level in pedicels. The *KNAT 3/4* genes belong to class II and were expressed in a wide variety of tissues, including floral organs (petals, stamen and sepals), cauline and senescing leafs. The *KNAT5/7* genes were primarily expressed in seeds. In *Populus*, the class I *KNOX* genes showed a higher and constant expression in roots, xylem, and seedlings, whereas the class II genes showed a more diverse expression pattern not only in root and seedlings but also in young leaves, female catkins. The class I genes in maize were mainly expressed in the shoot tip, stem and SAM, the internodes and immature tassel, while the class II genes were expressed in leaves, roots, and seedlings. In rice, the class I genes were expressed in inflorescences and pistils, and two of the class I genes were expressed in embryos. Class II genes in rice were expressed in seedlings, shoots, and leaves.

**Figure 6:**
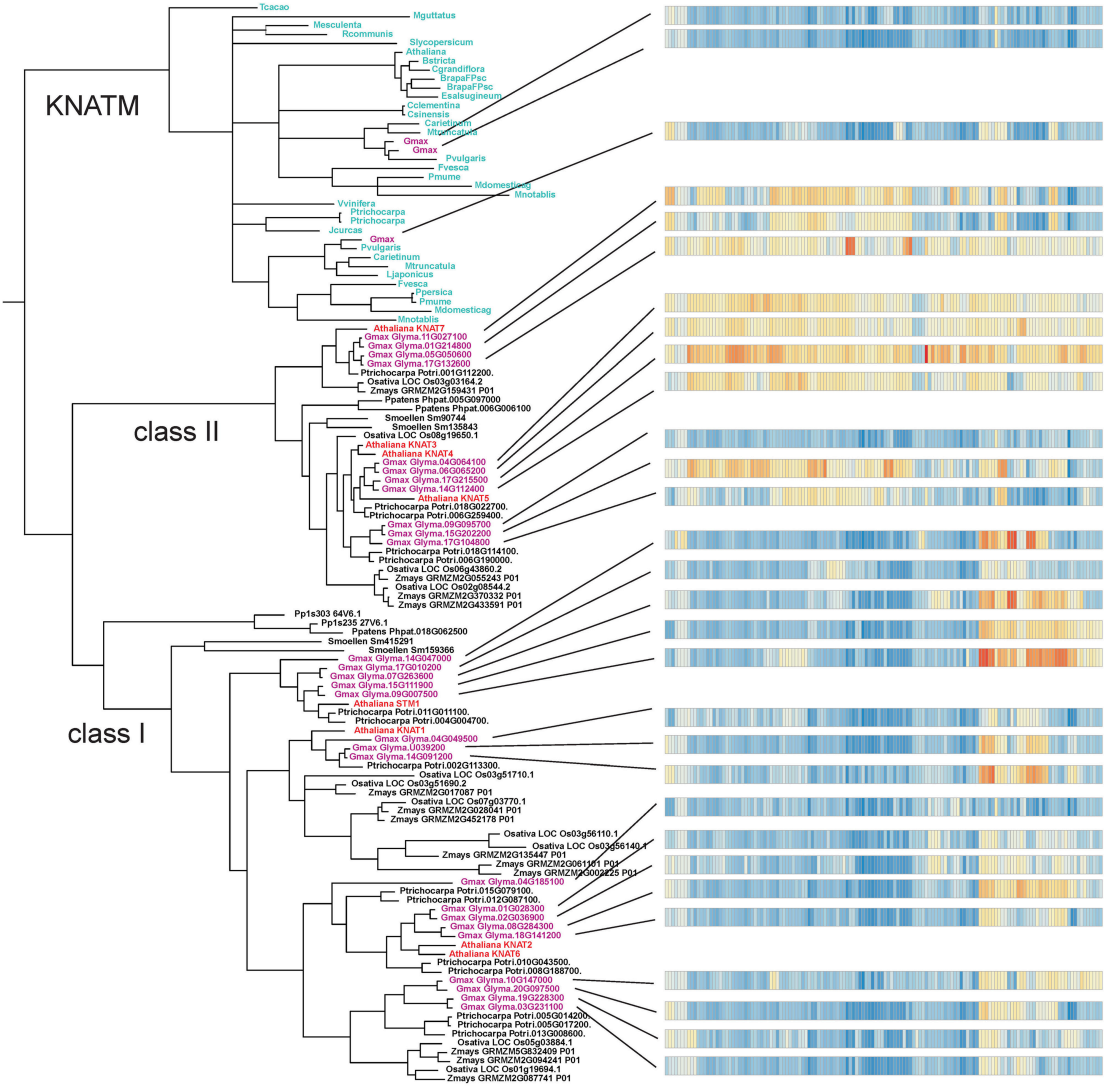
**Diversification and expression of ***Glycine max*** KNOX proteins**. Each horizontal bar on the right represent a set of 138 different RNA-Seq samples from the Harada-Goldberg soybean seed LCM dataset. RNA-Seq analyses are described under “Materials and Methods” and more details on this figure may be found in the Supplementary Tables 1, 2, Supplementary Data Sheet 1.

The soybean *G. max* has a large number of KNOX paralogs (Figure 1). To examine gene expression in this species we made use of a large RNA-Seq dataset (for details see under Section Materials and Methods). The results show that a large majority of the genes are expressed and have distinct tissue specificities (Figure 6, Supplementary Figure 7, Supplementary Table 2). In general, homologs of *KNAT3/4/5* and *KNAT7* (class II) tended to be expressed in a wide variety of tissues, whereas the class I members had a more specific pattern of expression. A majority of the class I genes were expressed in embryonic shoots and SAMs. In addition to these general observations we note that there are two *STM1* homologs in *G. max* that are strongly expressed in the SAM. At the same time, there are other *STM1* homologs that do not seem to be confined to SAM only. Furthermore, one of the *KNAT7* paralogs was strongly expressed in the hilum and hourglass of seed coat. Finally, three *KNATM* paralogs are overall poorly expressed. We observed the most significant expression for a *KNATM* gene located on chromosome 7, and its expression was strongest during the early maturation of axis plumules, followed by cotyledon vasculature, cotyledon stage embryo proper, and heart stage of the embryo.

## Discussion

Plant *KNOX* genes, first identified more than 20 years ago, play important roles in the formation and evolution of plant leaves (Hake et al., 2004; Furumizu et al., 2015; Tsuda and Hake, 2015). In this study, we identified a total of 394 KNOX proteins in green plants, including green algae. Our results demonstrated that the KNOX domain proteins are ubiquitous in the plant kingdom.

Our phylogenetic analysis supports the monophyly of the *KNOX* family, as well as monophyly of the class I and class II KNOX genes (Bürglin, 1997; Bharathan et al., 1999; Reiser et al., 2000). KNOX genes from green algae fall outside the two major subclasses. Thus, *KNOX* genes duplicated into two subclasses after the land plants diverged from algae and before the split between bryophytes and land plants 400 MYA ago (Gray, 1993; Kranz et al., 1995; Furumizu et al., 2015). It has been reported that the green algal KNOX gene from *Acetabularia acetabulum* exhibits a mixture of features common to the class I and class II *KNOX* genes in higher plants (Serikawa and Mandoli, 1999; Hay and Tsiantis, 2009). Here, we argue that the KNOX genes in green algae seem more related to the class I subclass, and class I proteins may therefore represent the ancestral form of KNOX. The most recent phylogenetic study of the KNOX and BELL genes showed that the divergence between these gene lineages occurred before the split between red and green algae and that a duplication in KNOX led to *KNOX I* and *KNOX II* genes in land plants (Furumizu et al., 2015). The presence of KNOX proteins in both prasinophytes and Ulvophyceae suggests that the KNOX proteins arose relatively early in the evolution of green algae and may have been lost in some phyla such as Chlorophyceae. Within the monophyletic KNOX family, KNATM represents a distinct clade as previously shown (Magnani and Hake, 2008). Our analyses revealed several previously unknown members of the KNATM family, which has many paralogs.

The *KNOX* genes have experienced two major events of expansion during the evolution of plants, one from algae to moss and the other during the transition from the lycophytes to angiosperms. Our study found that there was only one class of *KNOX* genes prior to the emergence of terrestrial plants, consistent with results from previous studies (Mukherjee et al., 2009; Furumizu et al., 2015). The green algae have only one *KNOX* gene but bryophytes possess five *KNOX* genes that are representatives of both classes I and II. After the first *KNOX* gene duplication, the *KNOX I* and *KNOX II* genes in moss have developed distinct functions. In our RT-PCR expression analysis, the five *KNOX* genes in *Physcomitrella* showed a differential expression pattern, but conclusions were limited by the restricted number of tissues we included. However, prior studies have used GUS-reporter genes to show the spatio-temporal expression patterns of the *KNOX* genes in moss, and these studies have revealed that the *KNOX I* and *KNOX II* genes are expressed throughout sporophyte development. *KNOX II* genes have also been detected in the haploid tissues, such as egg mother cells and mature eggs of mosses (Sakakibara et al., 2008, 2013). It was hypothesized that the *KNOX I* genes play a role in the maintenance of sporophytic meristematic cells, while *KNOX II* genes may regulate the gametophyte-to-sporophyte morphological transition (Sakakibara et al., 2008, 2013). Expansion of *KNOX* genes within mosses to include *KNOX I* and *KNOX II* paralogs may have been correlated with increasing fundamental structural and developmental challenges related to the transition to land (Raven, 1985, 1991; Edwards et al., 1986; Sztein et al., 1995).

The second expansion of *KNOX* genes occurred during the transition from lycophytes to angiosperms. Bryophytes and lycophytes have five and four *KNOX* genes, respectively, while the gene family is considerably larger and more diverse in angiosperms. Thus, we expect that the gene family expanded in angiosperms and experienced species-specific expansion in both eudicots and monocots. Angiosperms exhibit complex leaves with many specialized tissues compared to bryophytes, which do not have true leaves, and compared to lycopods, which possess simple microphylls. The complex, evolutionarily derived organ systems and structures of angiosperms might have required a larger number of KNOX proteins to maintain their existing biological requirements and to gain new functions. A number of important gene families involved in plant development have experienced gene expansion during angiosperm evolution. Examples are PGs (Polygalacturonase supergene family) (Yang et al., 2012) and some of the members of the transcription factor, such as the MADS-box gene family (Theissen et al., 2000), TCP transcription factors (Navaud et al., 2007), and NAC transcription factor (Hu et al., 2010). Therefore, it is not unexpected to observe an expansion of KNOX genes in angiosperms and it seems reasonable to assume that this expansion was a result of novel functional requirements. We have examined the chromosomal distribution of *KNOX* genes in selected species. We observed a number of tandemly duplicated genes (6/13) in rice (three pairs). On the other hand, we found no tandemly duplicated *KNOX* genes in the eudicots, *Arabidopis, Populus*, and *Glycine*. In the eudicots, we found that more *KNOX* genes were involved in large segmental duplication (data not shown). Our data suggest that tandem gene duplications may represent the major mode for rapid expansion of class I *KNOX* genes in rice. In contrast, segmental duplications are more likely to explain the expansion of *KNOX* genes in *Populus* and *Glycine*. In eudicots, segmental duplications are a consequence of whole genome duplication events, which are characteristic of the eudicot lineage (Tuskan et al., 2006; Schmutz et al., 2010).

The functions of KNOX TFs have diversified in diploid plants (Tsuda and Hake, 2015). The *STM* clade genes are observed only in eudicots. Therefore, it is possible that the *STM* originated at an earlier stage in the evolution of dicotyledons, and was lost in monocotyledons. In *Arabidopsis* and other higher plants, the *STM* gene is highly expressed in SAM (Elhiti et al., 2013; Stammler et al., 2013; Aguilar-Martinez et al., 2015). In *G. max*, two *STM* homologs are strongly expressed in the SAM and others are not specific to SAM. For the *KNAT1* gene, our data suggest that *KNAT1* in *Arabidopsis* and its homologs in maize are expressed in the stem, shoot apex and node, as well as the pedicels. Furthermore, there are three *KNAT1* homologs in *G. max* that are mainly expressed in early maturation axis SAM and axis vasculature. Previous studies suggested that *KNAT1* regulates internode development (Douglas et al., 2002; Venglat et al., 2002; Smith and Hake, 2003) and functions with *STM* in SAM maintenance (Byrne et al., 2002; Liebsch et al., 2014). *KNAT1* has also been reported to regulate inflorescence architecture (Douglas et al., 2002). Consistent with these results, we have also observed that *KNAT1* homologs in rice are highly expressed in inflorescences and pistils. *KNAT 2 and KNAT 6* homologs occur in other plants. In particular, the expression data of *G. max* showed that all the homologs of *KNAT 2/6* have similar expression profiles. Therefore, *KNAT 2/6* genes, at least in soybean, may be functionally redundant. It has previously been suggested that in *A. thaliana* the *ATH1*-*KNAT2* complex has a function overlapping that of *KNAT6* (Li et al., 2012a).

It is clear from our study that class II genes are characterized by broad tissue specificity compared to class I genes. Thus, class II genes are expressed not only in the leaves but also in floral organs, seeds, and roots. This diversified expression pattern may indicate that class II genes have evolved novel functions compared to class I genes. We have found that one of the class II *KNAT 7* paralogs in *G. max* is strongly expressed in the seed coat and hilum. Previous studies corroborate that *KNAT 7* is expressed in seed coat, as well as in the inflorescence stem in *Arabidopsis* (Li et al., 2011, 2012b). Additionally, previous studies have shown that *KNAT 7* interacts with MYB75 and other transcription factors in regulation of seed coat development. These prior studies predicted that *KNAT 7* might be involved in modulating secondary cell wall biosynthesis in both the *Arabidopsis* inflorescence stem and seed coat (Bhargava et al., 2013; Liu et al., 2014). Other studies also suggested that some members of this family—such as three of the *KNOX II* genes (*KNAT3, KNAT4*, and *KNAT5*) and one of the *KNOX I* genes (*KNAT1*)—are characterized by cell type-specific expression in the *Arabidopsis* root and may play roles in the regulation of root development (Truernit et al., 2006; Truernit and Haseloff, 2007). Furthermore, recent studies showed that *KNOX I* and *KNOX II* genes play opposite roles in regulating the development of all above-ground organs of the *Arabidopsis* sporophyte (Furumizu et al., 2015). In light of these results and our observation that class II proteins are widely expressed, conserved and under stronger purifying selection, we may speculate that this group of proteins have conserved functions in plant development yet to be discovered. Further functional studies are required to clarify the role of class II proteins.

The *KNATM* genes are expressed in an early phase of development in *A. thaliana*, and expression was observed in proximal-lateral domains of organ primordia and at the boundary of mature organs (Magnani and Hake, 2008). A functional analysis suggested the *KNATM* could dimerize with the *KNOX1* protein and play a role as a transcriptional regulator in leaf proximal-distal patterning (Magnani and Hake, 2008). A genetic study of a KNATM protein (*FCL1* gene) in *M. truncatula* indicated that it is indispensable in boundary separation and proximal-distal axis development of compound leaves (Peng et al., 2011). In our analysis of *G. max*, we have detected expression of the three *KNATM* paralogs. Of these, one is strongly expressed in early maturation axis plumules and showed less expression in cotyledon vasculature, cotyledon stage embryo proper, and heart stage embryo proper.

In conclusion, we have here used available protein data and bioinformatics methods, including profile HMMs, to better understand the evolution of the *KNOX* gene family. We show that the green algae proteins form a distinct clade in a phylogenetic analysis but seem most similar to the class I proteins of land plants. Proteins belonging to the KNATM family arose at a later stage during plant evolution as they are restricted to Eudicots. The *KNOX* genes have experienced two major events of expansion during the evolution of plants and specific plants are characterized by a dramatic increase in the number of *KNOX* paralogs. Consistent with previous studies, we observed for the higher plants that class II proteins have a relatively broad tissue specificity, whereas class I proteins have a much more restricted distribution. The patterns of gene expression in *G. max* and other plants presented here are detailed accounts of KNOX gene expression and they illustrate that expansion in gene number is reflected in functional diversity.

## Author contributions

JG and TS conceived and designed the research. JG conducted experiments. JG, WZ, LT, TS, and XY analyzed the data. GJ and TS wrote the manuscript. All authors read and approved the manuscript.

### Conflict of interest statement

The authors declare that the research was conducted in the absence of any commercial or financial relationships that could be construed as a potential conflict of interest.

## Supplementary material

Supplementary Table 1 **RT-PCR primers used to detect the expression of ***KNOX*** genes in ***Physcomitrella*** and ***Selaginella*****.

Supplementary Table 2 **Excel spreadsheet with ***G. max*** expression data**.

Supplementary Data Sheet 1 **Alignment of 394 KNOX protein sequences**.

Supplementary Figure 1 **Phylogram of KNOX proteins**. A phylogram version of the same data as in Figure 2.

Supplementary Figure 2 **Phylogenetic tree of KNOX gene families in selected species**. Tree was constructed using MrBayes as described in Section Materials and Methods. Posterior probabilities are shown at critical nodes.

Supplementary Figure 3 **Algae sequences group to classes I KNOX proteins—Alignment**. Alignment of selected KNOX proteins to illustrate relationship of the algae sequences to class I. Positions shared between algae and class I are highlighted with green arrows on top. Alignment in this figure has been simplified such that unreliable gapped regions in the original alignment have been removed.

Supplementary Figure 4 **Pairwise sequence identity of full-length ***KNOX*** proteins**. Species analyzed were *Physcomitrella patens, Selaginella moellendorffii, Oryza sativa, Zea mays, Arabidopsis thaliana, Populus trichocarpa*, and *Glycine max*. The box plot shows the median (black line), interquartile range (box), and maximum and minimum scores (whiskers) of each data set. Outliers are shown as circles outside of the whiskers.

Supplementary Figure 5 **Grouping of plant proteins in KNAT subclasses**. Phylogeny of selected plant species based on MrBayes using 84 KNOX protein sequences (The same tree was also used for PAML analysis). Species are *Physcomitrella patens, Selaginella moellendorffii, Oryza sativa, Zea mays, Arabidopsis thaliana, Populus trichocarpa*, and *Glycine max*. Nodes indicated with an orange circle have posterior probabilities in the range 0.5–0.9. All other nodes have posterior probabilities > 0.9.

Supplementary Figure 6 **Expression of the KNOX genes in ***Physcomitrella patens*** and ***Selaginella moellendorffii*****. The blue box indicates positive detection of gene expression in the corresponding tissues.

Supplementary Figure 7 *****Glycine max*** expression data**. The figure has the same data as in Figure 6, but with information on sample identity. See also Supplementary Table 2, an Excel spreadsheet of the quantitative data.

## Abbreviations

KNOX: homeodomain (HD) KNOTTED-like homeobox transcription factors
PGs: plant polygalacturonases
TCP: TCP transcription factors
NAC: NAM, ATAF1/2 and CUC2 domain proteins
SAM: shoot apical meristem
TFs: transcription factors.
