# Supplementary material for: Evolution, diversification, and expression of KNOX proteins in plants
Source: Front Plant Sci. 2015 Oct 23;6:882. doi: 10.3389/fpls.2015.00882 (PMC4617109; doi:10.3389/fpls.2015.00882)
Supplement: Supplementary Table 1 — RT-PCR primers used to detect the expression of KNOX genes in Physcomitrella and Selaginella. [file Table1.DOCX]

**Supplemental Table 1. RT-PCR primers used to detect the expression of *KNOX* genes in *Selaginella*.**

| **Genes** | **Primer name** | **Sequence (5'-3')** |
| --- | --- | --- |
| *Sm90744* | *Sm90744-SP1* | CCTCAGCAAAGACAAGGACGA |
|  | *Sm90744-SP2* | ACGTTGTATCGCCTGGAAGC |
| *Sm159366* | *Sm159366-SP1* | TTCATGGTTGCCTACTGTGCC |
|  | *Sm159366-SP2* | TCTGAAGGATTCCAATGACGCT |
| *Sm415291* | *Sm415291-SP1* | GCAATGTCCTCAACGCCTATG |
|  | *Sm415291-SP1* | GTGACCACCAGTCCAGCAGC |
| *Sm135843* | *Sm135843-SP1* | CTCAAGTCGTTCAAGGACCAGC |
|  | *Sm135843-SP2* | GAGTGTTCTCTCCGTCTCCGTG |
| *Sm_actin* | *Sm_actin-SP1* | TGGAGAAGATTTGGCACCATACT |
|  | *Sm_actin-SP2* | GGGAGTGCGTATCCTTCGTAG |
|  |  |  |
