# Supplementary figures and images for: Evolution, diversification, and expression of KNOX proteins in plants
Source: Front Plant Sci. 2015 Oct 23;6:882. doi: 10.3389/fpls.2015.00882 (PMC4617109; doi:10.3389/fpls.2015.00882)

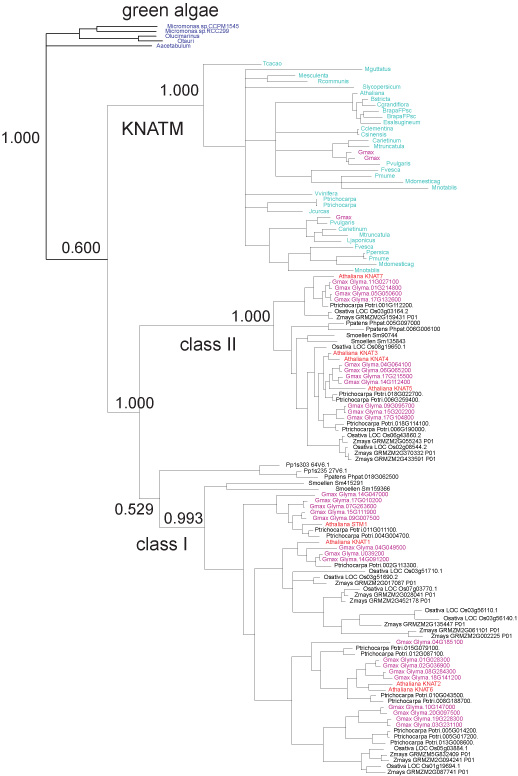

Supplement: Supplementary Figure 2 — Phylogenetic tree of KNOX gene families in selected species. Tree was constructed using MrBayes as described in Section Materials and Methods. Posterior probabilities are shown at critical nodes. [file Image2.JPEG]

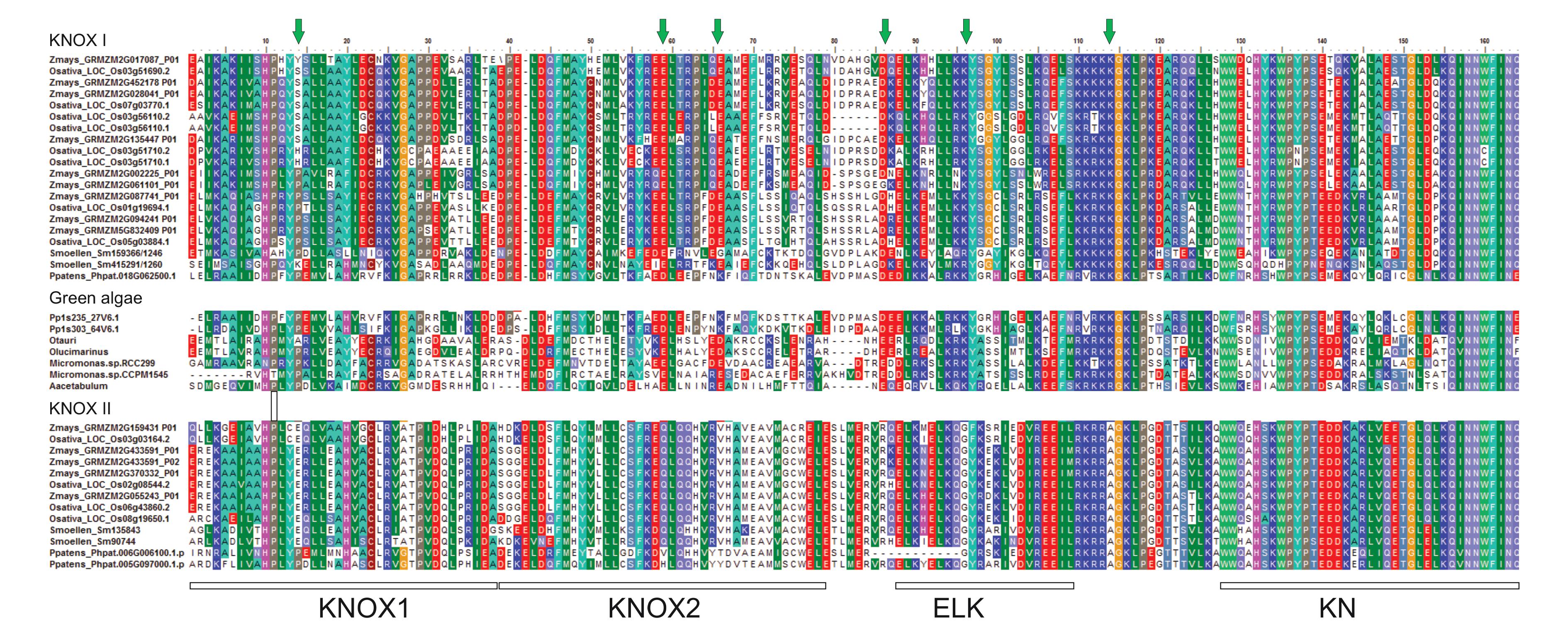

Supplement: Supplementary Figure 3 — Algae sequences group to classes I KNOX proteins—Alignment. Alignment of selected KNOX proteins to illustrate relationship of the algae sequences to class I. Positions shared between algae and class I are highlighted with green arrows on top. Alignment in this figure has been simplified such that unreliable gapped regions in the original alignment have been removed. [file Image3.JPEG]

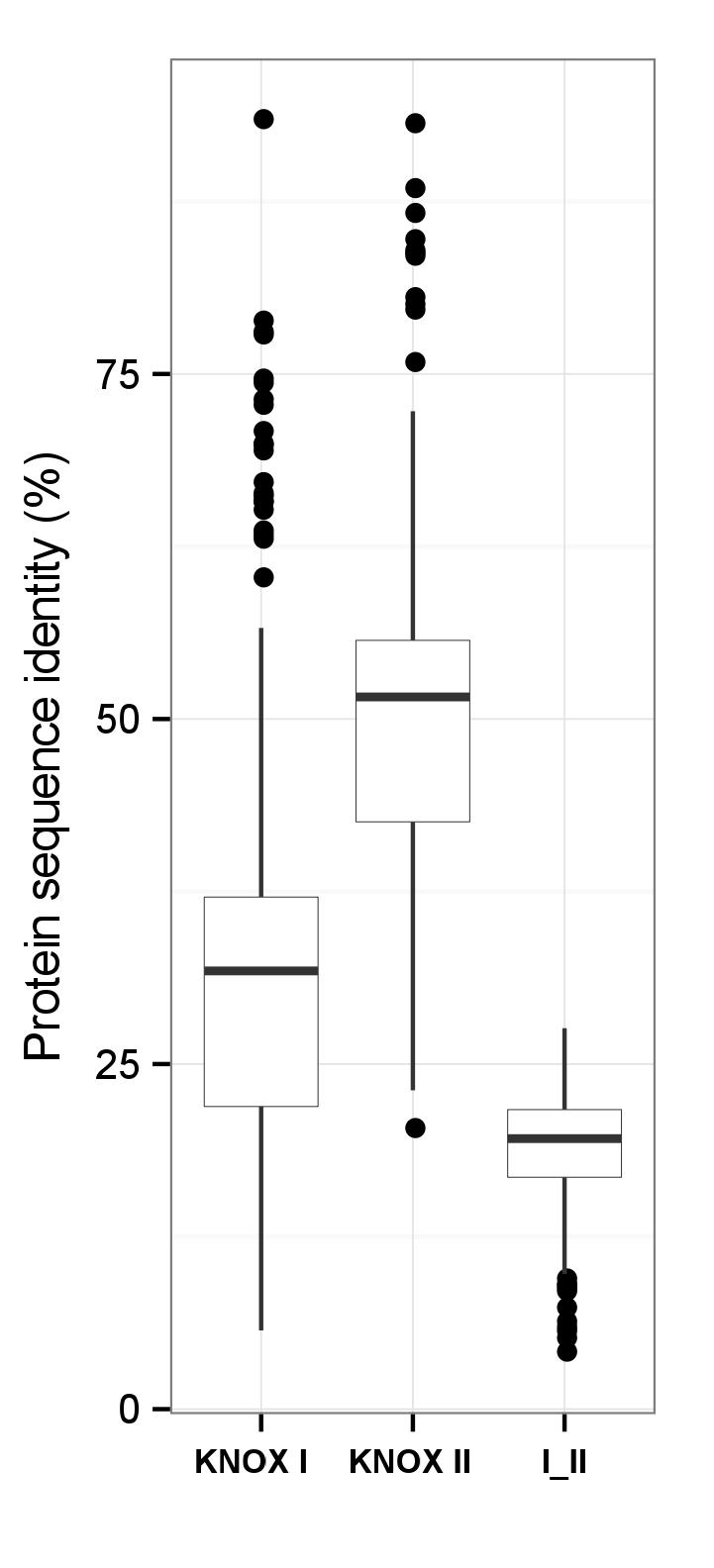

Supplement: Supplementary Figure 4 — Pairwise sequence identity of full-length KNOX proteins. Species analyzed were Physcomitrella patens, Selaginella moellendorffii, Oryza sativa, Zea mays, Arabidopsis thaliana, Populus trichocarpa, and Glycine max. The box plot shows the median (black line), interquartile range (box), and maximum and minimum scores (whiskers) of each data set. Outliers are shown as circles outside of the whiskers. [file Image4.JPEG]

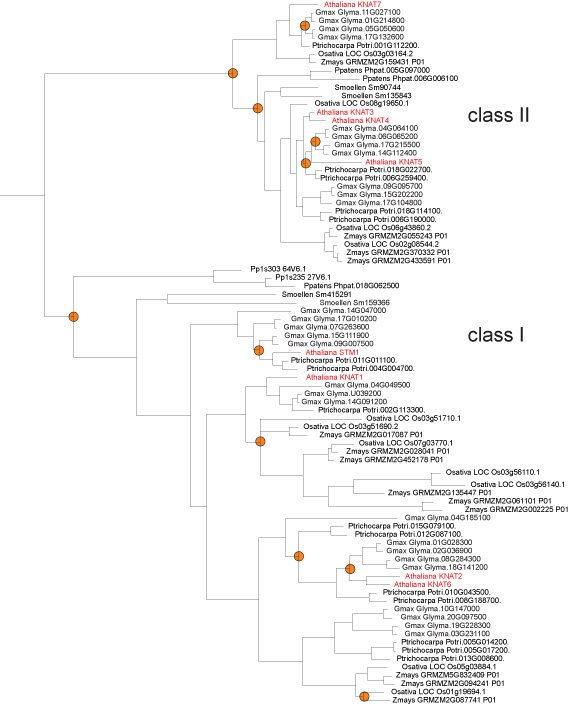

Supplement: Supplementary Figure 5 — Grouping of plant proteins in KNAT subclasses. Phylogeny of selected plant species based on MrBayes using 84 KNOX protein sequences (The same tree was also used for PAML analysis). Species are Physcomitrella patens, Selaginella moellendorffii, Oryza sativa, Zea mays, Arabidopsis thaliana, Populus trichocarpa, and Glycine max. Nodes indicated with an orange circle have posterior probabilities in the range 0.5–0.9. All other nodes have posterior probabilities > 0.9. [file Image5.JPEG]

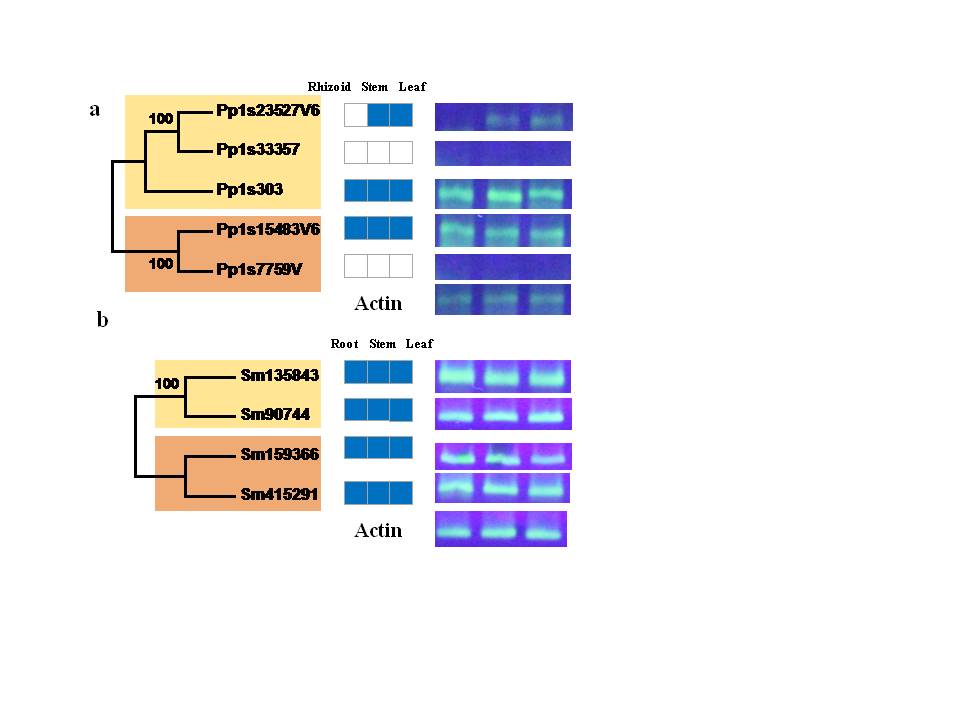

Supplement: Supplementary Figure 6 — Expression of the KNOX genes in Physcomitrella patens and Selaginella moellendorffii. The blue box indicates positive detection of gene expression in the corresponding tissues. [file Image6.JPEG]

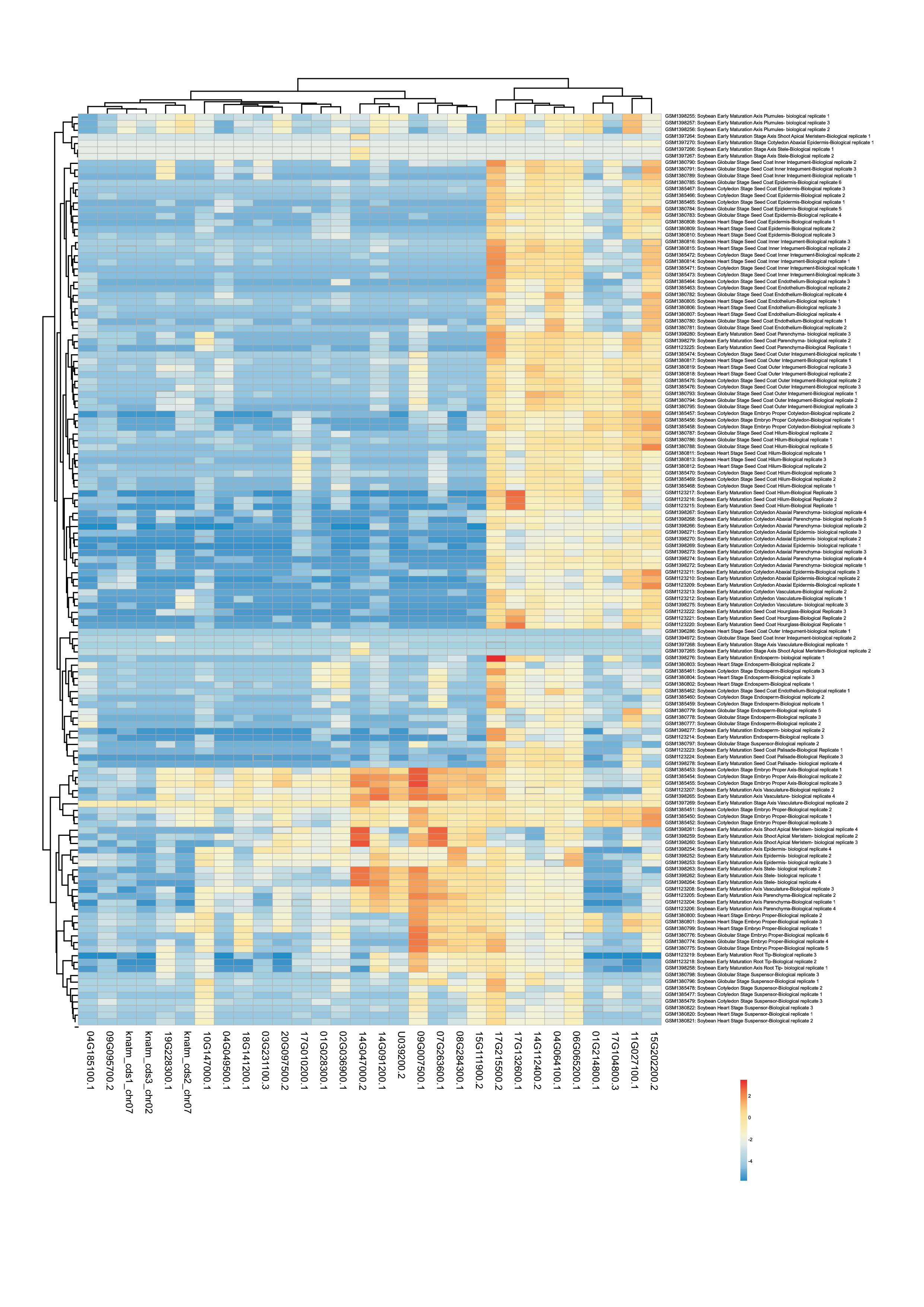

Supplement: Supplementary Figure 7 — Glycine max expression data. The figure has the same data as in Figure 6, but with information on sample identity. See also Supplementary Table 2, an Excel spreadsheet of the quantitative data. [file Image7.JPEG]
